# The Indigenous Birthing in an Urban Setting study: the IBUS study: A prospective birth cohort study comparing different models of care for women having Aboriginal and Torres Strait Islander babies at two major maternity hospitals in urban South East Queensland, Australia

**DOI:** 10.1186/s12884-018-2067-8

**Published:** 2018-11-01

**Authors:** Sophie Hickey, Yvette Roe, Yu Gao, Carmel Nelson, Adrian Carson, Jody Currie, Maree Reynolds, Kay Wilson, Sue Kruske, Renee Blackman, Megan Passey, Anton Clifford, Sally Tracy, Roianne West, Daniel Williamson, Machellee Kosiak, Shannon Watego, Joan Webster, Sue Kildea

**Affiliations:** 1 Midwifery Research Unit, Mater Research Institute-University of Queensland, Brisbane, QLD Australia; 2 School of Nursing, Midwifery and Social Work, University of Queensland, Brisbane, Australia; 3 Institute for Urban Indigenous Health, Brisbane, QLD Australia; 4 Aboriginal and Torres Strait Islander Community Health Service Brisbane Limited, Brisbane, QLD Australia; 5 Mater Misericordia Limited, Brisbane, QLD Australia; 6 The University of Sydney, Sydney, NSW Australia; 7 Griffith University, First Peoples Health Unit Queensland, Brisbane, Australia; 8 Department of Health, Aboriginal and Torres Strait Islander Health Branch, Brisbane, QLD Australia; 9 Australian Catholic University, Sydney, QLD Australia; 10 National Centre of Research Excellence in Nursing Interventions, Griffith University, Menzies Health Institute, Brisbane, QLD Australia

**Keywords:** Indigenous, Maternity, Midwifery, Health services research, Aboriginal and Torres Strait Islander, Health disparities, Birthing on Country, Prospective birth cohort, Preterm birth, Child mortality

## Abstract

**Background:**

With persisting maternal and infant health disparities, new models of maternity care are needed to meet the needs of Aboriginal and Torres Strait Islander people in Australia. To date, there is limited evidence of successful and sustainable programs. Birthing on Country is a term used to describe an emerging evidence-based and community-led model of maternity care for Indigenous families; its impact requires evaluation.

**Methods:**

Mixed-methods prospective birth cohort study comparing different models of care for women having Aboriginal and Torres Strait Islander babies at two major maternity hospitals in urban South East Queensland (2015–2019). Includes women’s surveys (approximately 20 weeks gestation, 36 weeks gestation, two and six months postnatal) and infant assessments (six months postnatal), clinical outcomes and cost comparison, and qualitative interviews with women and staff.

**Discussion:**

This study aims to evaluate the feasibility, acceptability, sustainability, clinical and cost-effectiveness of a Birthing on Country model of care for Aboriginal and Torres Strait Islander families in an urban setting. If successful, findings will inform implementation of the model with similar communities.

**Trial registration:**

Australian New Zealand Clinical Trial Registry #ACTRN12618001365257. Registered 14 August 2018 (retrospectively registered).

## Background

Maternal and infant health disparities have persisted between Aboriginal and Torres Strait Islander people and non-Indigenous Australians, with Aboriginal and Torres Strait Islander women having disproportionately higher rates of: maternal mortality (~ 3 times higher) [1]; preterm births (14% vs. 8%); low birth weight infants (liveborn: 12% vs. 6%); and perinatal deaths (12 vs. 9/1000) [2, 3]. Preterm birth is a leading cause of perinatal mortality, serious neonatal morbidity, and moderate to severe childhood disability [4]. It contributes to more than two-thirds of perinatal mortality (fetal loss and neonatal death) [5] and is associated with diabetes, cardiovascular and renal disease in adulthood [6]. Queensland [7] and Western Australian [8] research suggests the majority of perinatal deaths are associated with preterm birth and low birth weight, which in turn are linked to modifiable risk factors: maternal psychosocial stress [9], infections in pregnancy [10], smoking in pregnancy [11], limited maternal education and young maternal age [12]. An Australian Indigenous study found preterm births and perinatal deaths decrease as the number of antenatal consultations increases [13]. Targeting the antenatal period with interventions that are culturally safe and high quality are essential to addressing these health disparities [14–16].

### Birthing on Country

The Australian National Maternity Services Plan [17] identified three priority areas for improving services for Aboriginal and Torres Strait Islander women: 1) developing and expanding culturally competent maternity care; 2) developing and supporting an Aboriginal and Torres Strait Islander maternity workforce; and 3) developing dedicated programs for ‘Birthing on Country’ (best practice maternal and infant services for Aboriginal and Torres Strait Islander women).

An international literature review on Birthing on Country [14] was commissioned by the Australian government who defined Birthing on Country as:

‘*Maternity services designed and delivered for Indigenous women that encompass some or all of the following elements: are community based and governed; allow for incorporation of traditional practice; involve a connection with land and country; incorporate a holistic definition of health; value Indigenous and non-Indigenous ways of knowing and learning; risk assessment and service delivery; are culturally competent; and developed by, or with, Indigenous people*’ (p. 5).

The review assisted in identifying the characteristics of successful services [14] and at a national Birthing on Country workshop [18] participants described Birthing on Country as:

*‘a metaphor for the best start in life for Aboriginal and Torres Strait Islander babies and their families, an appropriate transition to motherhood and parenting for women, and an integrated, holistic and culturally appropriate model of care for all’* (p. 25).

Aboriginal participants described Birthing on Country as *‘the most powerful thing’* stating it was ‘*about cultural choice’* and ‘*being able to have babies safely on country’* (p. 33):

*‘not only bio-physical outcomes ... it’s much, much broader than just the labour and delivery ... (it) deals with socio-cultural and spiritual risk that is not dealt with in the current systems’* (p.24).

Workshop recommendations called for widespread system reform and the development of exemplar models of Birthing on Country in urban, rural and remote areas [18]. Guiding Principles for Developing a Birthing on Country Service Model and Evaluation Framework were subsequently developed and endorsed by the Australian Health Ministers Advisory Council based on the literature and a national workshop [19]. The key components of successful programs that should be integrated in Birthing on Country service models are shown in Table 1. To date, implementation and evaluation of such models has been limited.

**Table 1:** Key components of successful, culturally competent Birthing on Country Service Models, reproduced with permission [19]

Birthing on Country Maternity services designed by & delivered for Aboriginal & Torres Strait Islander women & families		
Governance Indigenous control, community development approach, shared vision cultural guidance & oversight		
Philosophy & Overarching Principles Respect for & incorporation of Indigenous knowledge & traditional practice; respect for family & mens’ involvement; partnership approach; women’s business; continuity of carer; connection with country/land; capacity building approach - particularly with training & education; holistic definition of health; choice; evidenced-based clinical practice; social model of health & wellbeing		
*Skill Acquisition, Training & Education* Partnership approach/ two-way learning; appropriately trained & supported; competency based; delivered on-site; career pathway from maternity workers to midwifery; health literacy for women & families	*Service Characteristics* Culturally competent service & staff; community based; specific location; designated ongoing funding; welcoming flexible service focusing on relationships & trust; outreach, transport, child friendly & group sessions; social, cultural, biomedical & community risk assessment criteria; clinical & cultural governance, interdisciplinary perinatal committee; effective IT; integrated services	*Monitoring & Evaluation* Designated funding for monitoring & evaluation; continuous quality assurance; audit activities & recall register
Results Community healing as evidenced by: reduced family separation at critical times, restoration of skills & pride; capacity building in the community; supporting community & family relationships; reduced family violence; increased communication & liaison with other health professionals & service providers; comprehensive, holistic, tailored care; improved maternal & infant health outcomes.		

### Caseload midwifery models

Continuity of care and carer has been identified as an important characteristic of culturally safe care for Aboriginal and Torres Strait Islander women [20]. Caseload midwifery (or midwifery group practice, MGP) is one such model that delivers continuity of midwifery carer throughout pregnancy, labour, birth and the early postnatal period [21]. MGP has been found to significantly improve outcomes for women and babies including reductions in preterm birth and increased satisfaction, breastfeeding, and cost savings [22, 23]. Only 10% of Australian women currently have access to caseload midwifery models, with only a disproportionately small number of Aboriginal and Torres Strait Islander women receiving this model of service delivery [24, 25]. Few services exclusively target Aboriginal and Torres Strait Islanderwomen and many women do not receive continuity of care across the maternity episode; therefore, there is a paucity of research in the area [26].

### The research gap

The main methodological limitations of published interventional studies aimed at improving care and services for Aboriginal and Torres Strait Islander mothers and babies are small sample sizes, short-term evaluations and a lack of an appropriate comparison group [14–16]. Although several studies show promising results, most lack appropriate methodology and/or statistical power. Birthing on Country is a complex intervention that has not yet been rigorously evaluated in an urban Australian setting. The current study will contribute to addressing this knowledge gap by employing a methodologically rigorous study design and participatory research methods to evaluate an urban Birthing on Country continuity of care model for Aboriginal and Torres Strait Islander families in Australia.

## Methods/Design

### Aims

This research project aims to evaluate the feasibility, acceptability, sustainability, clinical and cost-effectiveness, of maternity care for Aboriginal and Torres Strait Islander families in South East Queensland. Specifically, this study aims to:

1. Restructure services to incorporate the key characteristics of a Birthing on Country Service Model of maternity services for Indigenous women in an urban setting, including an enhanced midwifery group practice
2. Conduct an evaluation of the restructure using a prospective cohort study
3. Utilise participatory action research methods to ‘fine tune’ the restructure of services
4. Determine if women receiving this new model have improved maternal and infant health outcomes when compared to women receiving other models of care and baseline data
5. Determine the acceptability and sustainability of this new model of care
6. Evaluate the economic impact of this model
7. Explore the pregnancy and early parenting (six months postnatal) experiences of 20 women using an ethnographic approach accessing different models of care.

### Study setting

South East Queensland comprises one of the largest and fastest growing Aboriginal and Torres Strait Islander populations of Australia [27]. The Birthing in Our Community service, launched in 2013, is a new model of care informed by the Birthing on Country literature and Guiding Principles. This model of care is available to women having Aboriginal and Torres Strait Islander babies at the Mater Mothers’ Hospital (MMH), one of the largest tertiary maternity hospitals in Australia. Birthing in Our Community is conducted in partnership with two local Aboriginal Community Controlled Health Organisations: the Institute for Urban Indigenous Health (IUIH) and the Aboriginal and Torres Strait Islander Community Health Service Brisbane Limited (ATSICHS). The partnership is underpinned by a Memorandum of Understanding and Statement of Commitment to share resources to redesign maternal and infant health services. Service characteristics were based on the literature review [14] and the workshop report [18] as well as tailoring to the local context following recommendations from a World Café attended by 60 local stakeholders [28]; which resulted in the partnership. The overarching aim of the Birthing in Our Community partnership is to close the gap in Aboriginal and Torres Strait Islander maternal and infant health outcomes, particularly preterm birth, through the translation of evidence-based strategies into the Birthing in Our Community program.

In developing the evaluation of the Birthing in Our Community program we aimed to compare outcomes for mothers and infants with a similar cohort of women receiving standard care. After reviewing the statistics from across South East Queensland, the most appropriate comparison cohort was women attending the Royal Brisbane and Women’s Hospital (RBWH) where senior managers and researchers agreed to collaborate on a joint project and funding submission. However, as the funding was being awarded, the RBWH also changed their model of care (2013) for Aboriginal and Torres Strait Islander women through the Ngarrama Indigenous Maternity Service and it is no longer standard care. The Ngarrama Service is a government-funded midwifery continuity of care service for women having an Aboriginal and/or Torres Strait Islander baby/ies and planning to birth at the RBWH (located fewer than six kilometres north of the MMH). Thus we have agreed to evaluate both new services, Birthing in Our Community and the Ngarrama Service, and will compare them to each other, and to women receiving standard care and to baseline data from both hospitals. (Note: not all women having Aboriginal and Torres Strait Islander babies at both MMH and RBWH access the Indigenous-specific models of care hence the concurrent comparison with standard care). Table 2 outlines the components of the different models of care available to women having Aboriginal and Torres Strait Islander babies at the MMH and RBWH. For the first time, researchers will test the effectiveness of caseload midwifery in an urban setting where 100% of the midwives’ caseload are women having Aboriginal and Torres Strait Islander babies receiving care across the maternity continuum (antenatal, birth, until 6 weeks postnatal).

**Table 2:** Models of care available to Aboriginal and Torres Strait Islander families at study sites

Birthing in Our Community (Group 1)	Standard Care (Groups 2, 5)	Ngarrama Maternity Service (Group 4)
Indigenous governance (operating through a Steering Committee) functioning in accordance with Terms of Reference and underpinned by a MOU A community-based Midwifery Group Practice (MGP), which provides continuity of care to enrolled women throughout pregnancy, birth and up to six weeks postnatally Care is provided according to hospital guidelines and protocols in the home and at community venues where regular cultural and education days are held 24/7 access to caseload midwife who works in MGP of 4FTE on annualised salary with each woman allocated a primary midwife. Midwife support during birthing is likely to be by a known midwife. Location for birth is the Birth Suite (no homebirth or Birth Centre services provided)^a^ Indigenous Maternal Infant Health/ Family Support Workers and Indigenous student midwives work with the caseload midwives to provide culturally tailored care Referral to and integration with Indigenous community support agencies as required; All women are offered a formal handover to child health services with other referrals as required (e.g. paediatric, allied health) Clinical and cultural supervision for staff.	Antenatal care may be received from community based general practitioner, hospital based midwives or doctors who rotate throughout the service on rosters. Midwife support during birthing is likely to be by a midwife the woman has never met. Postnatal care or phone call from a rostered community midwife might take place if the woman meets the criteria for early discharge—before 48h for vaginal birth and 72h for caesarean section usually for up to less than two weeks No 24/7 access to MGP midwife but can call hospital birthing suite in emergency Location for birth same as other groups for the hospitals	A hospital-based Midwifery Group Practice (MGP) with a community clinic one day a week, which provides continuity of care to enrolled women throughout pregnancy, birth and up to six weeks postnatally. Care is provided according to hospital guidelines and protocols, regardless of setting (women’s homes, community venues or hospital) 24/7 access to MGP midwives who work in a small team and are on annualised salary with each woman allocated a primary midwife. Midwife support during birthing is likely to be by a known midwife. Location for birth may be in the Birth Suite or the co-located Birth Centre (eligibility criteria apply); Access to Cultural Capability Officers of the regional (Metro North) Aboriginal and Torres Strait Islander Health Unit who provide additional cultural guidance and support.
Indigenous Liaison Officers are based in the hospitals to strengthen culturally responsive care and support Access to medical staff, allied health professionals, social workers, child safety officers and other professionals (e.g. diabetic educator) as required Discharge letter to referral doctor and referrals to community support agencies as required		

^a^Despite community recommendations for an Indigenous Birthing Centre, funding has not yet been secured

### Study design

This study underwent peer review by the funding body, the National Health and Medical Research Council*.* This study will consist of a prospective cohort study comparing:

1. *Maternal and infant health outcomes*: women booked to receive maternity care through MMH or RBWH, by model of care and Aboriginal and Torres Strait Islander status of baby and/or mother (see Table 3). Routinely collected clinical outcomes for mothers and infants will provide data on the effectiveness of the programs since they commenced in late 2013 and be compared across groups for the duration of the study (ending in 2019), and to baseline data for both hospitals (2009–2013). Infant assessments will be undertaken at two and 6 months postnatally for Groups 1 and 4.
2. *Service acceptability, effectiveness and cost-effectiveness:* Maternal surveys will be undertaken as early as possible antenatally (~ 20 weeks and 36 weeks antenatally, two and six months postnatally). An ethnographic component will also explore the pregnancy and early parenting (to six months postnatal) experiences of ~ 20 women from each Birthing in Our Community and the Ngarrama Service using a longitudinal, ethnographic approach: ‘Tell My Story’ substudy. Cost-effectiveness analysis will be conducted from the broader ‘societal’ perspective which will include not only the cost to the hospital (Routine data) but the cost to the women (Women’s surveys) and partner organisations (Routine data).
3. *Service sustainability and feasibility:* Staff perspectives and experiences will be compared across the services to determine the sustainability and feasibility from a workforce perspective.

**Table 3:** Timeline of groups by model of care and research data available

Year	2009	2010	2011	2012	2013	2014	2015	2016	2017	2018	2019
Groups by model of care						Group 1: Birthing in Our Community, MMH (Indigenous)					
	Group 2: Standard care, MMH (Indigenous)										
	Group 3: Standard care, MMH (Non-Indigenous)										
						Group 4: Ngarrama Indigenous Maternity Service, RBWH (Indigenous)					
	Group 5: Standard care, RBWH (Indigenous)										
	Group 6: Standard care, RBWH (Non-Indigenous)										
Research data (Groups)	*Baseline*					*Study period*					
	Routine Data (2, 3, 5, 6)					Routine Data (1–6)					
						Women’s Surveys (1, 2, 4, 5)					
						Tell My Story (1, 4)					
						Infant Assessments (1, 2, 4, 5)					
						Staff Interviews (1, 4)					

Birthing in Our Community is a complex intervention with multifaceted components; identifying its ‘active ingredients’ is vital to evaluating effectiveness and replicating the intervention in other settings [29]. As with the Ngarrama Service, an appropriate monitoring and evaluation framework [29, 30] is needed that enables all stakeholders to understand not only what components are integral to success or failure but why these components are so important and influential. A Program Logic Model will be used for the monitoring and process evaluation to assess if the services are being implemented as planned [28]. Program logic is essentially a conceptual ‘road map’ that presents the thinking/theory behind the expected outcomes of research activities.

A mixed-methods research design has therefore been selected as the most appropriate to achieve these goals, with equal weight given to both qualitative and quantitative approaches. Incorporating participatory action research [31] (PAR), as recommended for Indigenous research [32], will allow the research team to be responsive to evaluation findings throughout the duration of the study. In line with a PAR approach, the IBUS research team will meet regularly to discuss relevant issues and collaboratively plan research-related activities, with regular reflections on previous steps, progress to date, and future expectations/aspirations [31]. Regular Steering Committee meetings provide a mechanism (Birthing in Our Community only) enabling timely feedback and regular reporting to key stakeholders.

### Study participants

#### Inclusion/exclusion criteria

##### Women’s surveys, Tell My Story and Infant Assessments

Women are eligible to participate if they:

- Are having an Aboriginal and/or Torres Strait Islander baby;
- Receive their maternity care through the Birthing in Our Community program and are planning to birth at the MMH (Group 1); receive their maternity care through the Ngarrama Indigenous Maternity Service and are planning to birth at the RBWH (Group 4); receive standard maternity care and birth at the MMH or RBWH (Groups 2 & 5); and
- Consent to participate.

Women are not eligible to participate if they:

- Have been transferred into the RBWH or the MMH from out of area for high-level specialist services or received no antenatal care.

Infants are eligible if:

- They are Aboriginal and/or Torres Strait Islander and their mothers received care through either of these hospitals and were recruited to the study.

##### Routinely collected clinical and costing data (Groups 1–6)

Women and infants will be excluded if they have been transferred into the RBWH or the MMH from out of area for high-level specialist services or received no antenatal care.

##### Staff surveys, interviews, and focus groups (Groups 1–6)

Staff are eligible to participate in the staff surveys, interviews and focus groups if they have been involved in the planning and/or provision of maternity care services for Aboriginal and Torres Strait Islander families in South East Queensland during the study period; and consent to participate.

#### Power and sample size

This study has been powered to detect changes in clinical outcomes and the number of women accessing care per annum in each program [33]. During the 3.5-year recruitment period, we will aim to access routinely collected data for approximately 420 women at MMH, and 350 from RBWH (based on an estimated 20% attrition rate). The change in outcomes has been estimated based on changes seen in the Townsville Mums and Bubs program that reported a reduction in preterm birth [34] (Table 4).

**Table 4:** Power calculations with *n* = 350 in each arm + 20% for attrition

Outcome	From %	To %	No./ cohort	Power
Proportion of women who attend ≥ 5 antenatal visits	81.0	90.0	261	0.924
Proportion of women who were smoking after 20 weeks	42.0	31.0	318	0.858
Preterm birth < 37 weeks pregnancy	16.0	9.0	350	0.801
Exclusive Breast feeding at discharge (2007–09 Mater data)	78.0	88.0	240	0.942

The sample size for the ethnographic component of the study (‘Tell My Story’) will involve a smaller group of women from each cohort. The project aims to recruit up to 25 women from each cohort, which factors in a 20% attrition rate (25*.8 = 20).

### Participant recruitment and informed consent

Written informed consent to participate in the study will be obtained from all participants. The privacy, wellbeing and safety of all participants is a paramount consideration, and will be ensured by strict adherence to eligibility criteria, ensuring the relevant Participant Information and Consent Form has been read and understood, and reminding participants of their right to withdraw from the study at any time, without penalty. Study data collection will not commence until the requisite site-specific Human Research Ethics Committee and Research Governance approvals have been secured.

#### Women’s surveys and Infant Assessments (Groups 1, 2, 4, 5)

Written information about the study will be provided to all women at both sites when they book into the hospital. Recruiting staff (midwives, health workers, and liaison officers) will fill out an ‘Expression of Interest’ referral form with women interested and willing for IBUS research staff to contact her to tell her more about the study. Women will then be contacted by IBUS research staff who will explain the study in more detail and invite them to participate. Women who agree to participate will be given, mailed or emailed the Participant Information and Consent Form, with verbal information provided by the research staff. Written informed consent to participate will be obtained by member/s of the research team who will meet women at their next antenatal visit or to ring them to discuss the study in more detail.

In line with the NHMRC *Values and Ethics Guidelines for Ethical conduct in Aboriginal and Torres Strait Islander Health Research* [32], women will be reminded that they may defer making a decision until they have had time to discuss the information with any “interested parties … formally constituted bodies … collectives or community elders” (p.14). Provision will also be made for answering any outstanding questions. Consent to participate will be obtained by member/s of the research team who will offer to meet them at their next antenatal visit or to ring them to discuss the study in more detail.

Although this study is not specifically targeting young women, we are guided by the National Statement in that they will not be excluded on the basis of age alone [35]. The study will also be guided by usual practice employed for clinical procedures whereby the best interests of the young woman, and her capacity to consent, will be assessed on an individual basis. Hence, young women “who are mature enough to understand and consent, and are not vulnerable through immaturity in ways that warrant additional consent from a parent or guardian” (p. 65) will be invited to consent in their own right [35]. Where there is any concern that immaturity renders a young woman vulnerable, she will not be invited to participate.

#### Routinely collected data (Groups 1–6)

We are seeking a waiver of consent to access routinely collected data for the all cohorts of women in order to satisfactorily answer the outcome measures. This will include both clinical and costing data. For women who participate in the IBUS surveys, consent will be sought to link IBUS survey data to routinely collected data. Initially the data will be collected in an identifiable form so we can be assured that data from different sources can be merged with each participant given one unique identifier. Once this is completed all identifiable data (e.g. name, address) will be removed and kept only in the participant log, which will be used to contact participants at different time points.

#### Tell My Story (Groups 1 & 4)

A smaller group of women will be invited to participate in the Tell My Story qualitative component. Women in the IBUS study who are interested in participating in further qualitative antenatal and postnatal follow-up will be approached by the IBUS research team.

#### Staff interviews and focus groups (Groups 1 & 4)

All staff members involved in planning or providing specialized maternity care to women having Aboriginal and/or Torres Strait Islander babies at MMH or RBWH will be eligible to participate in these individual and focus groups interviews. Staff will be invited to participate by the IBUS research team. A researcher will explain the staff interview process to the staff either face-to-face or on the phone using the Participant Information and Consent Form for Staff which will be emailed in advance. Staff who provide written consent to participate will be interviewed either annually or when exiting a role. Interviews will be conducted one-on-one or in small focus groups. Interview participants will be reimbursed for parking costs and given a small gift (e.g. chocolates) as a show of thanks and appreciation for their time.

#### Staff quantitative surveys (Groups 1–6)

Staff will be invited to complete surveys on their experiences of working in the different models of care. The initial invitation to participate will be sent to staff via email with the option for them to complete electronic surveys via an email link or paper surveys distributed via team leaders from the research team. Staff will receive face-to-face, text and email reminders to complete the survey.

### Data collection

#### Routinely collected clinical and costing data

Data for each mother/infant dyad at the MMH will be collected from several sources. MMH obstetric (MatriX), neonatal database and expenditure data (Australian activity based funding Diagnosis Related Groups [DRG] codes) will provide detailed patient-level information on inpatient contacts for the mother and baby. Routinely collected data including expenditure data will be collected from the RBWH where it will be coded before being merged with the study database. Perinatal data will also be extracted from the Queensland Health database.

The IUIH (MMeX) and ATSICHS (Medical Director and Pracsoft; MMeX) routinely collect data on service delivery (access, clinical data, e.g. immunisations, and expenditure data) which will also be collected. The clinical and costing outcomes data will be derived from routinely collected information and survey data.

#### Women’s surveys and infant assessments

Survey data collected at different time points will be used to measure program acceptability, sustainability and effectiveness; and infant growth and development. Women will be invited to complete face-to-face, postal or online surveys (their choice) at booking-in, 36 weeks of pregnancy, at two months and six months after birth. Maternal surveys will include questions related to women’s maternity care experience and out-of-pocket costs incurred in accessing maternity or child health services (see Table 5 for list of survey items). At two and six months after the birth, women will be asked to provide information about their infant’s development (Ages and Stages Questionnaire [36]). At six months postnatal, women and infants will be invited to participate in a face-to-face developmental assessment, and offered a developmental report on their infant’s performance on the Bayley-III Scales of Infant and Toddler Development [37]. Referral to a specialist will be available for infants identified with developmental delays or who otherwise raise concerns. Additionally, if any woman is identified to be at risk of depression or psychological distress or self-harm, she will also be offered referral to appropriate services.

**Table 5:** Items included in Women’s antenatal and postnatal surveys

Items	Women’s surveys			
	1	2	3	4
Socio-demographic characteristics				
Indigenous status, maternal and paternal	✓			
Maternal relationship status	✓			
Educational attainment, maternal and paternal	✓			
Employment status, maternal and paternal	✓			
Government pension main source of income	✓		✓	✓
Has healthcare concession card	✓			
Has private health insurance	✓			
Has access to vehicle/transport	✓			
Number of places of residence during pregnancy		✓		
Current housing	✓	✓		
Experienced homelessness during pregnancy		✓		
Financial insecurity	✓	✓	✓	✓
Self-reported health problems				
Experienced health problems, mother and infant			✓	✓
Baby admitted to hospital, date, reason, duration			✓	✓
Mother admitted to hospital, date, reason, duration			✓	✓
Number of visits with baby to child health nurse, reason			✓	✓
Number of visit with baby to paediatrician & reason			✓	✓
Pregnancy, birth/labour & care				
Gestation/age of baby	✓	✓	✓	✓
General feelings about pregnancy	✓	✓	✓	✓
Number of weeks first contact with care for pregnancy	✓			
Where was care received	✓			
Plans for birth location	✓			
Experience of staff behaviour		✓	✓	
Culturally safe aspects of care (importance, satisfaction)	✓		✓	
Felt respected & understood by hospital staff (by area)			✓	
Felt treated poorly or judged by staff			✓	
Satisfaction with care, recommend to others		✓		
Known midwife present during labour/birth			✓	
Attendance of group antenatal classes		✓		
Smoking				
Current smoking status, including number of cigarettes per day	D	✓	✓	✓
Attempts to quit	D	✓		
Advised to quit by health staff		✓		
Smoking support received, perceptions		✓		
Smoking household members	✓	✓		✓
Feeding baby				
Previous breastfeeding experiences, inc. difficulties	D	✓		
Intentions to breastfeed, inc. duration	D	✓		
Confidence to breastfeed		✓		
Experience of breastfeeding, inc. initiation			✓	
Use of formula, inc. reasons, age of baby			✓	
What baby has been fed in past 24h			✓	✓
Where received feeding information			✓	
Use of commercial baby food				✓
Reasons for starting solids				✓
Partner involvement				
Partner’s feelings about pregnancy	✓			
Baby’s father & father figures (involvement, attended visits, support services)				✓
Social and emotional wellbeing				
Negative life events - full extended version	✓	✓		✓
Additional worries experienced	✓	✓		✓
Family separations			✓	
Positive wellbeing scale	✓	✓	✓	✓
Modified Kessler Psychological Distress Scale (K5)	✓	✓	✓	✓
Edinburgh Postnatal Depression Scale	D			✓
Practical Social Support Scale	✓			
Social support available, inc. partner		✓		
Out-of-pocket costs				
Time spent, support person, carer, transport, food & drink		✓		
All services accessed for pregnancy/birth/baby, number of visits, out of pocket cost per visit (i.e. not refunded by Medicare)			✓	
Out of pocket cost of medicines during pregnancy/birth/baby			✓	
Infant development				
Ages and Stages Questionnaire			✓	✓
Bayley III Cognitive, Language and Motor Skills (face-to-face assessment)				✓

Note: D = clinical data available

Participants will be provided with an AU$10 gift card after completing the booking-in and 36 weeks antenatal surveys and a large tote bag at 36 weeks. An AU$30 gift card and a small toy or bib/blanket will be given for the two and six-month postnatal surveys to thank women for their time. Women who participate with their infant in the Bayley assessment will receive an additional AU$10 gift card upon completion.

#### Tell My Story

Researchers will interview women and discuss family practices including lifestyle, stressors, social support, cultural practices and childrearing. There will be the option for women to do a one-off in-depth interview or to take part in repeated interviews antenatally and until the infant is six months old. Interviews will explore Indigenous perspectives of culturally safe care (acceptability) and what constitutes social, cultural and clinical risk and wellbeing for Aboriginal and Torres Strait Islander women. Women will be asked about their relationships with healthcare providers, their experiences with maternity services and how these impact engagement, health choices and outcomes.

Interviews will be recorded using a digital recorder and observations recorded using hand written notes. Antenatal and postnatal interviews will be conducted in a convenient location for women – in the home, clinic or hospital. Participants will be provided with a AU$30 gift voucher as a thank-you for their time.

#### Staff surveys, interviews and focus groups

##### Staff interviews and focus groups

All staff who have provided specialised maternity care for women having Aboriginal and/or Torres Strait Islander babies at either the MMH (including Birthing in Our Community) or at the RBWH (including the Ngarrama Indigenous Service) will be invited to participate in staff interviews and focus groups. This includes but is not limited to exit interviews with staff leaving or who have left the service. These interviews will explore the experiences of staff working within the programs and identify any recommendations for future and existing services. This will assist with identifying the ‘key ingredients’ of a best practice model of maternity care as well as evaluating the acceptability, feasibility, and cost-effectiveness of the different models of care. Semi-structured interviews will be conducted over the phone, in person or in small groups. With the staff members’ permission, these interviews will be audio-recorded.

##### Staff surveys

In order to evaluate the sustainability of the models from a workforce perspective, annual quantitative surveys will be conducted with staff involved in the program. A comparison group of MMH and RBWH caseload midwives will also be invited to participate in selected surveys to assess whether there is a significant difference in workload, daily activities and work-related stress between the caseload midwifery teams. Online and anonymous surveys will be conducted with staff. These will be voluntary and confidential, and likely include the Maslach Burnout Inventory or Copenhagen Burnout Inventory [38], the Attitudes to Professional Role, Caseload Midwifery Industrial Agreement Questionnaire, Time in Motion Study, Kessler Psychological Distress and Wellbeing Scale as well as questions about team cohesion, meeting goals, cultural capability of staff, and suggestions for improvements.

### Outcome measures

#### Primary outcome measures (all groups)

- Proportion of women giving birth preterm (< 37 weeks gestation)
- Proportion of women who attend five or more antenatal visits during pregnancy
- Proportion of women smoking after 20 weeks gestation
- Proportion of women exclusively breast-feeding at discharge from hospital.

#### Secondary outcome measures (all groups where reliable data is available)

- Gestation at first antenatal visit to a health provider, at booking into hospital (weeks, Mean, Median, Range, First trimester (Yes/No)), Number of total antenatal visits (Mean, SD, Median, Range, < 5 visits, 5 and more visits)
- The proportion of women with modifiable risk factors for preterm birth (i.e. inadequate antenatal care, smoking, stress and missing data in these fields)
- Smoking status at booking (Yes/No), during the first 20 weeks (Yes/No), and after the first 20 weeks (Yes/No), at discharge and six months postnatal, Number of cigarettes intake each day if smoking (Mean, SD)
- The proportion of women who attended antenatal education sessions (Yes/No)
- Pharmacological analgesia in labour (Epidural/spinal analgesia, Narcotic analgesia, Nitrous oxide gas)
- Onset of labour (Induced, No labour, Spontaneous)
- Mode of birth (Non-instrumental vaginal birth, Instrumental vaginal birth, Elective Caesarean section, Emergency Caesarean section)
- Management of third stage labour (Active, Physiological)
- Postpartum haemorrhage (< 500; 500–999; 1000-1499; 1500 ml and more or with blood transfusion)
- Perineal trauma status (Intact/1st degree tear, 2nd degree tear, 3rd/4th degree tear).
- Episiotomy (Yes/No)
- Women who had a known caregiver for labour and birth (Yes/No)
- Birth weight (grams, Mean, SD, < 2500 g, 2500 g or more)
- Apgar score 5 min (< 7, 7 or above)
- Admission to a separate neonatal nursery (Yes/No)
- Perinatal outcomes (Liveborn survived, Liveborn neonatal death prior to discharge from hospital, Stillbirth)
- Cause of perinatal deaths
- Antenatal intention to breastfeed (Yes/No)Women exclusively breastfeeding at discharge from hospital following birth (Yes/No), at two months postnatal (Yes/No), and six months postnatal (Yes/No)
- Mother readmission to hospital up to six months postpartum (Yes/No)
- Infant readmission to hospital up to six months of age (Yes/No)
- Length of stay in hospital for mothers and infants following birth (Mean, Median, Range)
- Cost of care per mother/infant pair during pregnancy, birth, postnatal until mother six weeks postpartum and baby 28 days after birth
- Negative life events scale – full extended version – at booking-in and six months postnatal (Yes/No, Number of events)
- Modified Kessler Psychological Distress Scale (K5) score at booking-in, 36 weeks gestation, two and six months postnatal
- Edinburgh Postnatal Depression Scale score at booking-in and six months postnatal
- Ages and Stages Questionnaire score at two and six months postnatal
- Bayley III Cognitive, Language and Motor Skills score at six months postnatal

Note, all tools will be scored according to their recommended guidelines and outcomes reported accordingly.

### Data analysis and management

#### Quantitative

Clinical data will initially be collected in a re­identifiable form (via the unique patient identifier) so that it can be linked with data obtained from other sources (e.g. surveys), to enable the economic analyses to be undertaken. Once merged, identifiers will be removed and only the coded number will remain.

Quantitative analyses will compare the difference in clinical outcomes between Birthing in Our Community (intervention), the Ngarrama Service (concurrent control), standard care (historical and concurrent control), and also non-Indigenous women and babies (historical and concurrent control) at MMH and RBWH.

Data on all women attending either hospital during the study period will be extracted. Women transferring in from other hospital or rural and remote areas for higher-level services (variable available), and those with no antenatal care will be excluded from analysis. Analyses will be by birth model of care and women with multiple births and their infants with identified fetal anomaly will be excluded from analysis unless noted otherwise.

Initial bivariate analysis will investigate possible differences between the cohorts for baseline socio-demographic (socio-economic status), and clinical characteristics (e.g. age, parity, body mass index, smoking, obstetric history) that could affect outcome measures. Dependent on data type, analysis will be undertaken using an independent samples t-test, Mann-Whitney U test or chi-squared test. Outcome measures will be presented using relative risks with 95% confidence intervals. Multivariate logistic, linear regression models and propensity score matching will be used to adjust for confounders. Longitudinal outcomes (e.g. breastfeeding) will be analysed with generalized estimating equations to account for the correlation between observations repeated in the same person. To understand the mechanism or process that underlies the effect of model of care on outcomes, mediation analysis will be conducted to identify if and to what extent the other variables explains the relationship. All withdrawals, losses to follow-up, and deaths will be reported. Analysis will be performed with SPSS Version 22.0/Stata 14.0 and statistical significance will be at the 0.05 level.

Survey data will be uploaded from iPads/tablet computers, Qualtrics or other software tools such as Remark (for the printed versions) to a dedicated spreadsheet and subjected to simple descriptive analysis using SPSS/Stata. Bayley-III data at the six-month infant assessment will be scored by the research assistant in real time (i.e. as the assessment proceeds) using the age-standardized Bayley Record and Score Forms, and associated Tables to give norm-referenced scores. These scores will then be uploaded onto the project specific database. Staff surveys will be analysed using SPSS/Stata to test for significant trends and potential differences between groups.

#### Cost-effectiveness analysis

The cost-effectiveness analysis will be conducted to examine the direct costs, from a societal perspective, to women and their families, maternity and child health care services and other community services in relation to pregnancy and birth. We will compare the mean costs per mother/infant pair between Birthing in Our Community (Group 1) and the Ngarrama Service (Group 4) to standard care group (Group 2 and 5) up to 6 weeks postpartum. Costs will be calculated for both mother and baby to include: women and family’s out-of-pocket expenses related to clinic appointments, outpatients (ultrasound, pathology, etc.) and prescribed medicines, and hospitalisation costs. Data will be collected through routinely collected information as well as questions embedded in the 36-week antenatal and 2 month postnatal surveys. Average costs for each mother and infant for the duration of the maternity episode (i.e. from when she first confirmed her pregnancy to 6 weeks postnatal) will be calculated and compared to determine the cost effectiveness of a model of care.

#### Qualitative

Qualitative interview data from the Tell My Story study will be audiotaped, as will staff focus groups and interviews. Data will be analysed using Interpretative Phenomenological Analysis (IPA), a qualitative method of analysis which draws knowledge from everyday experiences and is descriptive. Transcripts will be read by a minimum of two team members who will identify key themes and independently create a coding system. Codes will be compared, inconsistencies discussed and reconciled, and a final coding scheme agreed before entry into NVivo Version 8. The analysis will comprise of: Firstly, coding of initial interview transcripts to identify themes and develop a coding framework. Secondly, identification and categorisation of women and staff experiences using the coding framework. The coding framework will be revised and refined as new themes emerge. Finally, key findings will be used to inform the development future Birthing on Country services. Data will be saved on the computer hard drive and transcribed verbatim.

## Discussion

This study will test the impact of two maternity models of care for one of Australia’s highest priority health populations: Aboriginal and Torres Strait Islander mothers and babies. The model and economic impact assessment have been derived from international best-practice and service evaluations in Australia and our study has the statistical power to detect a difference in preterm birth. The multi-agency approach to implementing a Birthing on Country Service Model at one site has been recommended in Aboriginal and Torres Strait Islander policy documents and will be evaluated. The economic impact assessment aims to quantify the impact of the model by articulating the process by which research leads to impacts on the end-user and/or the broader community. If the model is successful and demonstrates a good return on investment, we will have developed and evaluated a culturally safe service model transferable to other settings for trialling in a broader context.

## Abbreviations

ATSICHS: Aboriginal and Torres Strait Islander Community Health Service Brisbane Ltd
BiOC: Birthing in Our Community
IBUS: Indigenous Birthing in an Urban Setting
IUIH: Institute for Urban Indigenous Health
MMH: Mater Mothers’ Hospital
NHMRC: National Health and Medical Research Council (Australia)
RBWH: Royal Brisbane & Women’s Hospital

## References

[1] Australian Institute of Health and Welfare: Maternal deaths in Australia 2012–2014. *Cat. No. PER 92*. Canberra: AIHW; 2017.

[2] Australian Institute of Health and Welfare: Australia Mothers and Babies 2015 - In Brief. *Perinatal* statistics *series no 33*, Cat. no. PER 91. Canberra: AIHW; 2017.

[3] Li Z, et al: Australia’s mothers and babies 2010. Canberra: AIHW National Perinatal Epidemiology and Statistics Unit; 2012.

[4] Gotsch F (2009). The preterm parturition syndrome and its implications for understanding the biology, risk assessment, diagnosis, treatment and prevention of preterm birth. J Matern Fetal Neonatal Med.

[5] Lumley J, Chamberlain C, Dowswell T, Oliver S, Oakley L, WatsonL. Interventions for promoting smoking cessation during pregnancy. Cochrane Database Syst Rev. 2009;(3):CD001055. https://www.ncbi.nlm.nih.gov/pmc/articles/PMC4090746/. 10.1002/14651858.CD001055.pub3 PMC4090746 19588322

[6] Gluckman PD, Hanson M, Pinal C (2005). The developmental origins of adult disease. Maternal & Child Nutrition.

[7] Johnston T, Coory M (2005). Reducing perinatal mortality among Indigenous babies in Queensland: should the first priority be better primary health care or better access to hospital care during confinement?. Australian and New Zealand Health Policy.

[8] Freemantle C (2006). Patterns, trends, and increasing disparities in mortality for Aboriginal & non-Aboriginal infants born in WA, 1980-200. Lancet.

[9] Moutquin J (2003). Socio-economic and psychosocial factors in the management and prevention of preterm labour. BJOG.

[10] Sangkomkamhang U, et al. Antenatal lower genital tract infection screening and treatment programs for preventing preterm delivery. Cochrane Database Syst Rev. 2008:CD006178. 10.1002/14651858.CD006178.pub3 PMC8498019 25922860

[11] British Medical Association (2004). Smoking and reproductive life: the impact of smoking on sexual, reproductive and child health.

[12] Muglia L, Katz M (2010). The enigma of spontaneous preterm birth. N Engl J Med.

[13] Steering Committee for the Review of Government Service Provision: Overcoming Indigenous disadvantage: key indicators. Canberra: steering Committee for the Review of government service provision, Productivity commission; 2009.

[14] Kildea S, Van Wagner V: ‘Birthing on country,’ Maternity service delivery models: a review of the literature. Canberra: maternity services inter-jurisdictional Committee for the Australian Health Minister’s advisory council, brokered by the sax institute; 2013.

[15] Herceg A: Improving health in Aboriginal & Torres Strait Islander mothers, babies and young children: a literature review. Canberra: Australian Government Department of Health & ageing; 2005.

[16] Rumbold A, Cunningham J (2008). A review of the impact of antenatal care services for Australian Indigenous women and attempts to strengthen these services. Matern Child Health J.

[17] AHMAC: National maternity services plan, 2011. Canberra: Australian health ministers advisory council, Commonwealth of Australia; 2011.

[18] Kildea S, Stapleton H, Magick Dennis F: Birthing on Country Workshop Report, Alice Springs, 4th July. Brisbane: ACU and Mater Medical Research Institute; 2013.

[19] Kildea S, Lockey R, Roberts J, Magick Dennis F: Guiding Principles for Developing a Birthing on Country Service Model and Evaluation Framework, Phase 1. Brisbane: Mater Medical Research Unit and the University of Queensland on behalf of the Maternity Services Inter-Jurisdictional Committee for the Australian Health Ministers’ Advisory Council; 2016.

[20] Kruske S: Culturally competent maternity Care for Aboriginal and Torres Strait Women Report. Brisbane: September 2012 – prepared on behalf of the maternity services inter-jurisdictional Committee for the Australian Health Ministers’ advisory council; 2013.

[21] Hartz D, Foureur M, Tracy SK (2012). Australian caseload midwifery: the exception or the rule. Women and Birth.

[22] McLachlan HL (2012). Effects of continuity of care by a primary midwife (caseload midwifery) on caesarean section rates in women of low obstetric risk: the COSMOS randomised controlled trial. BJOG.

[23] Tracy Sally K, Hartz Donna L, Tracy Mark B, Allen Jyai, Forti Amanda, Hall Bev, White Jan, Lainchbury Anne, Stapleton Helen, Beckmann Michael, Bisits Andrew, Homer Caroline, Foureur Maralyn, Welsh Alec, Kildea Sue (2013). Caseload midwifery care versus standard maternity care for women of any risk: M@NGO, a randomised controlled trial. The Lancet.

[24] Homer C (2012). It’s more than just having a baby’ women’s experiences of a maternity service for Australian Aboriginal and Torres Strait islander families. Midwifery.

[25] Stamp G (2008). Aboriginal maternal and infant care workers: partners in caring for Aboriginal mothers and babies. Rural Remote Health.

[26] Kildea S, Tracy S, Sherwood J, Magick-Dennis F, Barclay LM (2016). Improving maternity services for Indigenous women in Australia: moving from policy to practice. Med J Aust.

[27] Biddle N: CAEPR Indigenous population project: 2011 census papers. Canberra: Centre for Aboriginal Economic Policy Research, Australian National University; 2013.

[28] Kildea Sue, Hickey Sophie, Nelson Carmel, Currie Jody, Carson Adrian, Reynolds Maree, Wilson Kay, Kruske Sue, Passey Megan, Roe Yvette, West Roianne, Clifford Anton, Kosiak Machellee, Watego Shannon, Tracy Sally (2018). Birthing on Country (in Our Community): a case study of engaging stakeholders and developing a best-practice Indigenous maternity service in an urban setting. Australian Health Review.

[29] Campbell N (2007). Designing and evaluating complex interventions to improve health care. BMJ.

[30] Craig P, et al.: Developing and evaluating complex interventions: new guidance. Medical Research Council; 2008. 10.1136/bmj.a1655 PMC2769032 18824488

[31] Stringer E (1999). Action Research 2nd Edition.

[32] NHMRC (2003). Values and Ethics: Guidelines for Ethical Conduct in Aboriginal and Torres Strait Islander Health Research.

[33] Kildea S, Stapleton H, Murphy R, Kosiak M, Gibbons K (2013). The maternal and neonatal outcomes for an urban Indigenous population compared with their non-Indigenous counterparts and a trend analysis over four triennia. BMC Pregnancy and Childbirth.

[34] Panaretto K (2007). Sustainable antenatal care services in an urban Indigenous community: the Townsville experience. Med J Aust.

[35] National Statement on Ethical Conduct in Human Research. The National Health and Medical Research Council, the Australian Research Council and universities Australia. Commonwealth of Australia, Canberra; 2007 (updated 2018).

[36] Squires J, Twombly E, Bricker D, Potter L (2009). ASQ-3 User's guide.

[37] Bayley N: Bayley scales of infant and toddler development, third edition (BAYLEY-III). Pearsons Clinical; 2005.

[38] Kristensen T, Borritz M, Villadsen E, Christensen K (2005). The Copenhagen burnout inventory: a new tool for the assessment of burnout. Work & Stress.

[39] NHMRC (2005). Keeping research on track: A guide for Aboriginal and Torres Strait Islander people about about health research ethics.

